# Statement on falls in long‐term care facilities by the Japan Geriatrics Society and the Japan Association of Geriatric Health Services Facilities

**DOI:** 10.1111/ggi.14332

**Published:** 2022-01-19

**Authors:** Hiromi Rakugi, Ken Sugimoto, Hidenori Arai, Koichi Kozaki, Yasumoto Matsui, Katsuyoshi Mizukami, Yasumasa Ohyagi, Jiro Okochi, Masahiro Akishita

**Affiliations:** ^1^ Department of Geriatric and General Medicine Osaka University Graduate School of Medicine Suita Japan; ^2^ Department of General and Geriatric Medicine Kawasaki Medical School Okayama Japan; ^3^ National Center for Geriatrics and Gerontology Obu Japan; ^4^ Department of Geriatric Medicine Kyorin University School of Medicine Tokyo Japan; ^5^ Center for Frailty and Locomotive Syndrome, National Center for Geriatrics and Gerontology Obu Japan; ^6^ Graduate School of Comprehensive Human Sciences, Faculty of Health and Sport Sciences University of Tsukuba Tsukuba Japan; ^7^ Department of Neurology and Geriatric Medicine Ehime University Graduate School of Medicine Toon Japan; ^8^ Geriatric Health Services Faculty Tatsumanosato Daito Japan; ^9^ Department of Geriatric Medicine, Graduate School of Medicine The University of Tokyo Tokyo Japan

**Keywords:** falls, long‐term care facility, geriatric syndrome

## Abstract

In current clinical practice, when a fall occurs in a long‐term care facility, it is often treated as an accident. Falls are classified as one of the most commonly prevalent geriatric syndromes. As their causes are extremely diverse and complex, their occurrence rate depends on individual susceptibility, even if appropriate fall prevention measures are taken. Falls are common among older adults, and fractures and intracranial hemorrhage resulting from falls can lead to the deterioration of activities of daily living and death. For this reason, it is recommended that the risk of falls is assessed in the general population of older adults, and that appropriate interventions are carried out for those at high risk. In response to this situation, the Japan Geriatrics Society and the Japan Association of Geriatric Health Services Facilities have issued the following statements on falls as a geriatric syndrome based on scientific evidence, especially considering the frequent occurrence of falls in long‐term care facilities. **Geriatr Gerontol Int 2022; 22: 193–205**.

**Statement 1: Not all falls are accidents caused by negligence.**

**For facility residents who are at high risk of falling, even if fall prevention measures are implemented, falls will occur at a certain rate. Even if fractures or trauma occur because of falls, they cannot necessarily be regarded as accidents caused by negligence in medical and nursing care settings.**

This is a principle that should be widely shared not only with those directly involved, such as long‐term care facility (LTCF) administrators and staff, residents and their families, but also with the public. The standard measures for fall prevention might change in the future because of new research on fall prevention, but for the time being, the current measures will probably be continued. It is important to clarify this point in advance; for instance, at the time of admission, rather than after a fall has occurred. It is recommended that the understanding of this specific point is reached when explaining the prognosis of new residents.

Although a fall in an LTCF does not necessarily imply negligence, it is necessary to establish a system to verify the circumstances under which the fall occurred and to utilize this information for subsequent fall prevention.

**Statement 2: In principle, care and rehabilitation should be continued.**

**Care and rehabilitation to maintain and improve the daily living functions of residents might increase the risk of falls because of the accompanying increase in activity. However, in many cases, the maintenance and improvement of daily living functions are expected to preserve and improve quality of life. Therefore, in principle, they should be continued.**

Rehabilitation and encouragement of daily exercise for residents is beneficial in terms of maintaining and improving their daily living functions. For example, it is known that the longer the time spent out of bed during the day, the better activities of daily living are preserved in older people who require nursing care.^1^ In contrast, a characteristic of nursing home residents is that they will experience falls at a certain rate if they are active. It is necessary to continue efforts to maintain and improve the daily living functions of residents based on their comprehensive assessment, and not to focus exclusively on falls, and fall‐related injuries and deaths.

**Statement 3: Ensure risk awareness and educational interventions among LTCF staff and residents, their families, and other concerned parties in the understanding that falls are a geriatric syndrome.**

The information that should be shared among the concerned parties includes the following: the current risk of falls; the risk of fractures and death caused by falls; the existence of standard fall prevention measures; the limited effectiveness of fall prevention measures as people get older; falls being one of the geriatric syndromes; fracture and death caused by falls being common in people at high risk of falling, in the natural course of geriatric syndromes; the current status of fall prevention measures in each LTCF; the procedures for responding to falls; and, finally, the fact that physical restraints are not used, in principle, in facilities, and the reasons behind this choice. For reference, Table 1 shows the information that should be shared with LTCF staff, residents and their families from the time of admission to the time of discharge.

**Table 1:** Examples of assessments and explanations regarding falls (examples of information that should be shared with long‐term care facility staff, residents and their families)

1. Conduct fall risk assessment at the time of admission
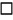 Fall risk assessment and countermeasures in the LTCF care plan (at admission and periodic review)
2. Provide explanations to residents and their families (at the time of admission and additionally as necessary)
Results of fall risk assessment
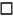 Risk of falling due to deterioration of health or decline in activities of daily living at and during admission
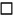 Increased risk of falling due to the change in the environment by moving to a LTCF
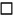 In some cases, the risk of falling increases as mobility improves with rehabilitation and treatment
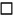 No physical restraint in principle and the reasons for not doing so
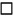 Fall prevention measures implemented in LTCF
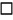 Items to keep in mind for you and your family
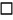 The mechanism of falls and the existence of a certain probability that a fall will occur, even if fall prevention measures are taken. The possibility of falls is especially high in people with a high risk of falling.
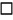 The possibility of fracture or intracranial hemorrhage due to a fall, which might lead to deterioration of daily functions and affect life.
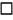 LTCF response procedures in the event of a fall (e.g. computed tomography imaging in the event of head injury, response in the event of bone fracture)
3. Review of medical care by physicians to prevent falls, fall‐related fractures and deaths
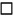 Antiplatelet agents and anticoagulants
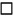 Sleeping pills, anxiolytics, antipsychotics and antidepressants
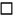 Antihypertensive and hypoglycemic drugs
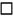 Polypharmacy
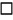 Indications for osteoporosis drugs
As a rule, #1 and #2 should be carried out in a multidisciplinary manner.

LCTF, long‐term care facility.

**Statement 4: Establish measures to prevent falls and countermeasures in case of falls, and review them periodically.**

**In addition to fall prevention measures, facilities should develop appropriate response procedures in the event of falls, make them known to staff, and explain them in advance to residents, their families and other concerned parties. Although there are no standardized measures recommended for nursing homes at this time, scientific evidence and technology continue to advance; hence, facilities should periodically review fall‐related measures and procedures.**

Each LTCF should clearly state its specific approach and policy regarding fall prevention measures. At present, there is no uniform standard protocol for fall prevention in facilities, but it is important to clarify the fall prevention measures in each LTCF, and explain them in writing to residents and their families, both in advance and regularly. General measures include assessment of the risk of falls, exercise (rehabilitation) and multifaceted interventions for modifiable risks, such as the reviewing of medications.

As shown in Statement 2, despite implementing these fall prevention measures, falls occur at a certain rate. Therefore, facilities should prepare a response procedure (in the form of a written manual), tailored to the current situation in each LTCF, to appropriately respond to the occurrence of falls. Table 2 shows an example of a response procedure.

Although it is generally considered acceptable that fall prevention measures and procedures for responding to fall incidents will not be at the same level as those used in hospitals, various factors will have an impact on these measures and procedures, including scientific evidence and technological advances regarding fall prevention in LTCFs, the preferences of residents and their families, and the status of cooperation with medical institutions in the vicinity of the LTCF. A periodic review is recommended, depending on each LTCF. Information that is continuously being published and revised, such as the Cochrane Database of Systematic Reviews^2^ described below and the "White Paper on Fall Prevention" by the Japanese Society for Fall Prevention,^3^ is useful for this purpose.

**Table 2:** Example of response procedures in the event of falls (including falling down and falling off)

1. Identification of the injured person's medical condition by the person who discovered the injury
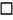 Status of the injured area (trauma and fracture)
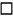 Level of consciousness, response to voice commands, response to instructional actions
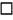 Subjective symptoms (headache, nausea, pain on movement)
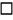 Other findings (vomiting, left–right difference in pupils, paralysis)
2. Reporting and information sharing with relevant parties
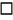 Report to the physician (facilities with a supervising physician)
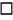 Contact family as soon as possible
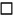 Detailed fall records
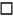 Information sharing among staff members
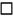 Notification to the municipality (in the case of fractures)
3. Doctor's response
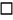 Determining the need for emergency transport
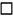 Determining the need for radiography
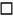 Determining the need for head computed tomography scan
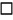 Determining the need to discontinue antithrombotic drugs
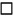 Setting the time for follow up

## Basic knowledge of falls as a geriatric syndrome

**Figure 1:**
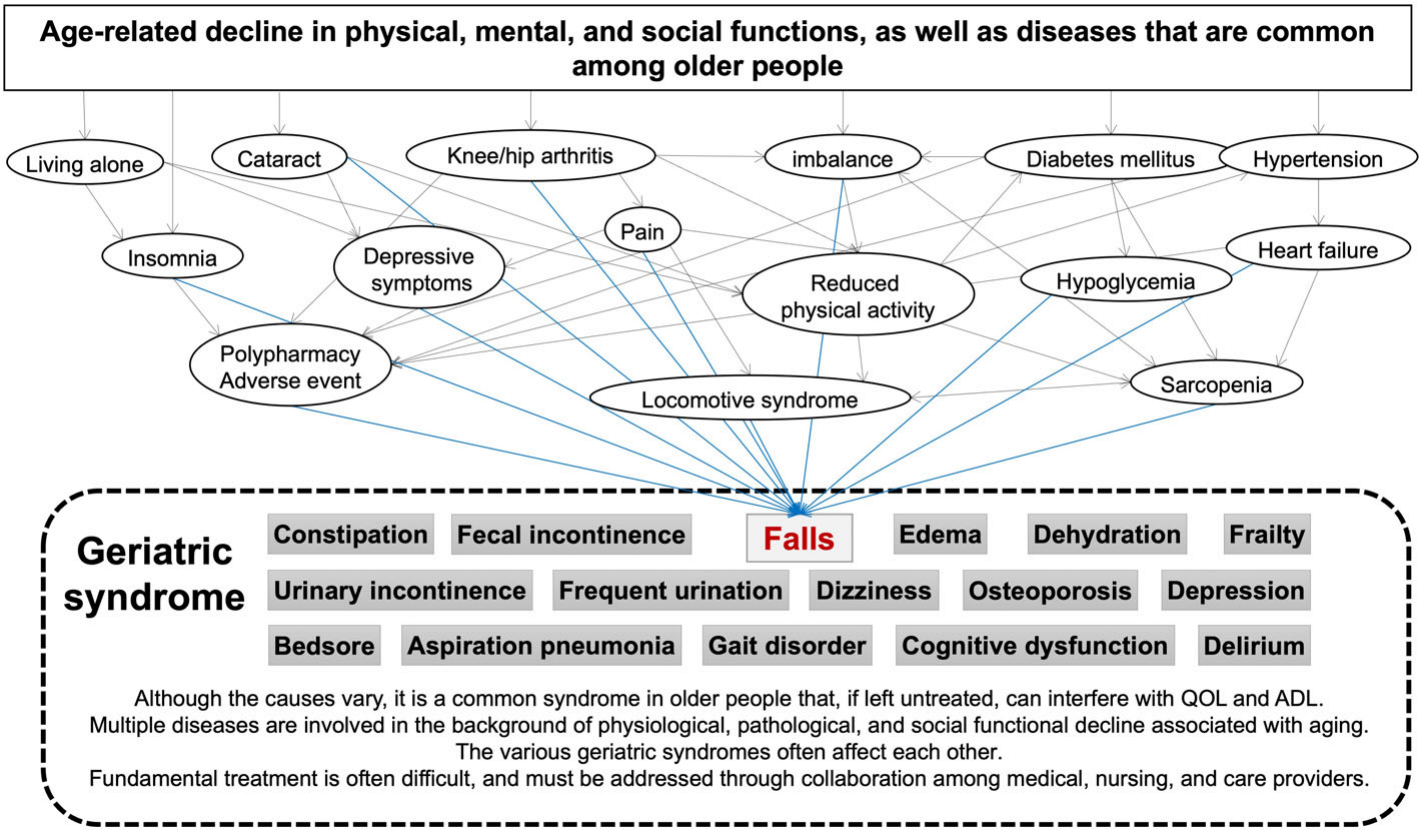
The concept of geriatric syndrome. The arrows show falls as an example; however, many factors are also involved in other syndromes. Each functional decline or disease causes multiple syndromes rather than a specific disease. Each syndrome among the geriatric syndromes often interacts with other geriatric syndromes, and the syndromes enclosed by dashed lines can correspond to the items represented in the oval boxes above. Many of the oval items are indeed also classified as geriatric syndromes.

The concept of geriatric syndrome is shown in Figure 1. Geriatric syndrome is a general term for a variety of syndromes that are frequently observed in old age, such as falls, urinary incontinence, bed sores and delirium. It is a complex condition that does not fall into any individual disease entity, and is often complicated by multiple underlying factors, such as the deterioration of multiple organ functions, psychological factors, environmental factors, social factors and use of medications. It is called a “syndrome” because multiple symptoms often appear in a sequence or in relation to each other. Falls are one of the most common geriatric syndromes, and the preface to the Guidelines for the Prevention of Falls in Older People (in Japanese) states that *"Falls in older adults are a disease, not an accident,” and “… should understand falls as a ‘disease’ or ‘syndrome’ caused by physical factors rather than an accident."* ^4^

**Figure 2:**
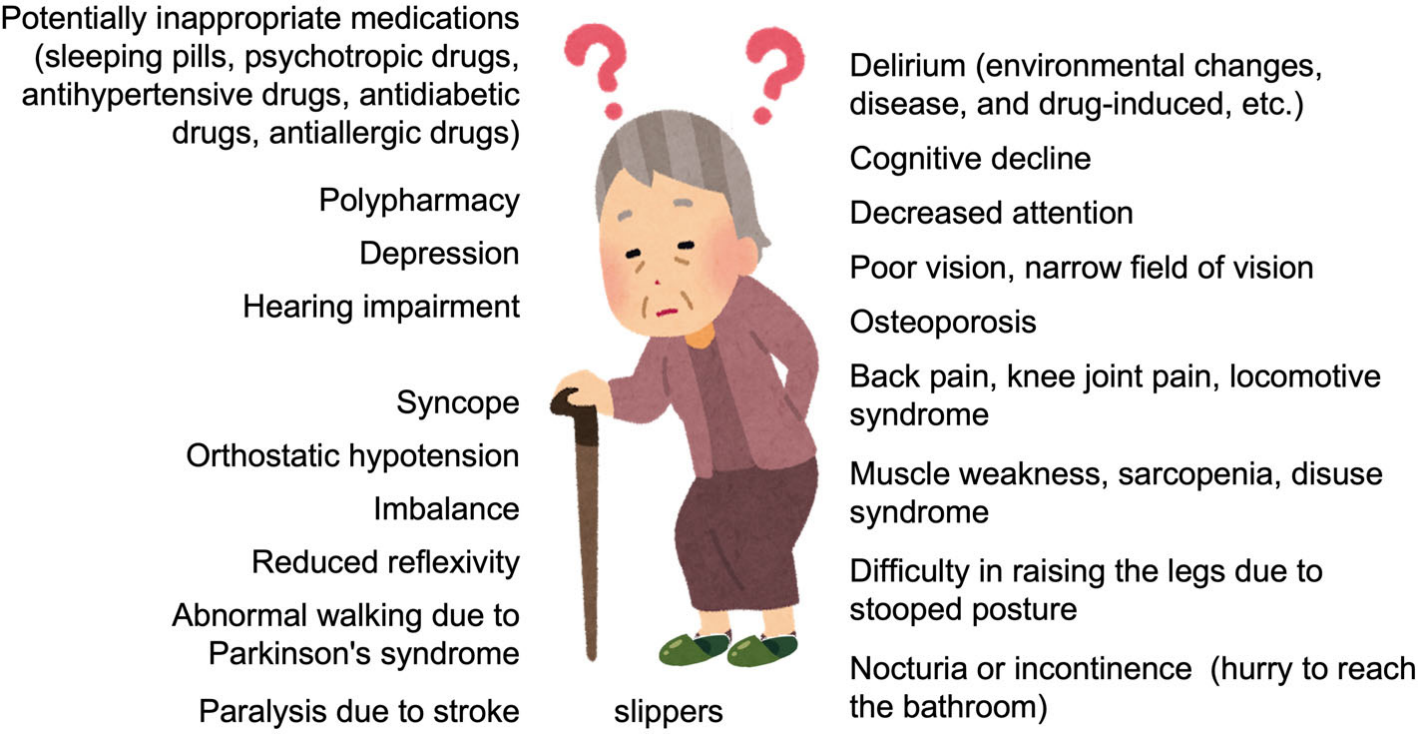
Common complex risk factors for falls and fractures among nursing home residents. The incidence of falls in many institutionalized residents is due to a combination of multiple factors that often interact with each other. Many falls occur in the early stages of institutionalization owing to changes in the environment and a lack of familiarity with toileting care. Another risk factor is when fewer people are involved in looking after the residents at the long‐term care facility than at home before admission. Except for the risk associated with slippers, many factors are difficult to modify or are progressive. The introduction of rehabilitation to improve symptoms and increase activity might increase the chance of falling. Injuries due to falls are also more likely to occur because the residents no longer live on tatami mats, unlike at home.

Two main factors contribute to the occurrence of falls in older adults: individual (internal) factors and environmental (external) factors.^5^ The risk of individual factors increases with age due to disease progression and decline in physical function. Figure 2 shows a situation in which various factors related to falls with aging are superimposed on a single individual. Clearly, this condition is common in facility residents, and many factors are difficult to improve, even through medical intervention. Environmental factors vary and generally include slippery floor surfaces, coarse carpets, frayed carpets, unsecured obstacles, inadequate or defective household items, poor lighting and uneven transitions, such as level changes between rooms; facilities have implemented countermeasures for these factors. Environmental factors in facilities include the change in the environment itself on admission, unfamiliarity with facilities and the need for assistance during defecation.

## Fall prevention and the procedure to follow when a fall occurs

There are many environmental factors related to the risk of falling that can be addressed, and proactive measures are being taken in facilities for older adults. In general, measures to prevent falls are extremely important for older adults in terms of preserving individual daily living functions and life prognosis.

The World Health Organization published the “Integrated Care for Older People: Guidelines on community‐level interventions to manage decline in intrinsic capacity,” which provides the following considerations and recommended measures regarding fall prevention.^6^

*Falls are the leading cause of hospitalization and injury‐related death. Extrinsic factors include environmental hazards, such as loose rugs, clutter, poor lighting, and improper footwear including ill‐fitting, floppy slippers. Intrinsic factors include abnormalities in any of the organ systems that contribute to postural control, such as the sensory, musculoskeletal, and central nervous system. Older people can decrease their fall risk with exercise, physical therapy, home‐hazard assessments and adaptations, and withdrawal of psychotropic medications*.

- *Medication review and withdrawal (of unnecessary or harmful medication) can be recommended for older people at risk of falls. (Quality of the evidence: low; strength of the recommendation: conditional)*
- *Multimodal exercise (balance, strength, flexibility, and functional training) should be recommended for older people at risk of falls. (Quality of the evidence: moderate; strength of the recommendation: strong)*
- *Following a specialist's assessment, home modifications to remove environmental hazards that could cause falls should be recommended for older people at risk of falls. (Quality of the evidence: moderate; strength of the recommendation: strong)*
- *Multifactorial interventions integrating assessment with individually tailored interventions can be recommended to reduce the risk and incidence of falls among older people. (Quality of the evidence: low; strength of the recommendation: conditional)*

Although fall prevention measures are recommended, it should be noted that, among older adults, and especially in the old‐old population, the factors that cause falls tend to be more multifactorial, complex and severe; hence, there are more falls that cannot be prevented by environmental maintenance or other measures, or that cannot be treated by managing the individual diseases.

Although the “Analysis of Deaths Related to Head Injuries Caused by Falls and Falling During Hospitalization,” published in June 2019 by the Medical Accident Investigation and Support Center of the Japan Medical Safety Research Organization, is a survey analysis specifically focused on hospitalized patients, the following statement is generally important^7^:

*Falls are one of the so‐called geriatric syndromes that occur in older adults due to various causes. Therefore, it is impossible to prevent them completely. However, with the aging of the population, risk assessment and countermeasures related to falls and falling should be implemented, and that appropriate measures should be taken if there is head trauma after a fall or falling*.

The same principle applies to facilities. However, *“measures related to falls”* and *“appropriate responses”* in the case of a head injury after a fall differ in various ways in facilities from those that can be used in hospitals. For example, in hospitals, there is a recommendation to *“consider head computed tomography (CT) scan after a fall, even if there is no obvious abnormality, depending on the situation”*. However, it is necessary to make a comprehensive judgment for each LTCF and each resident, by considering the medical resources of the LTCF; the availability of nearby medical institutions; and the health status, preferences and prognosis of the residents. This should also include their cognitive functions; and considerations for surgical treatment in cases wherein a head CT scan shows abnormality. Therefore, it is recommended that each LTCF determines in advance its policy and approach on how to respond based on the health condition of the residents.

## Current status of care and death related to falls in Japan

**Figure 3:**
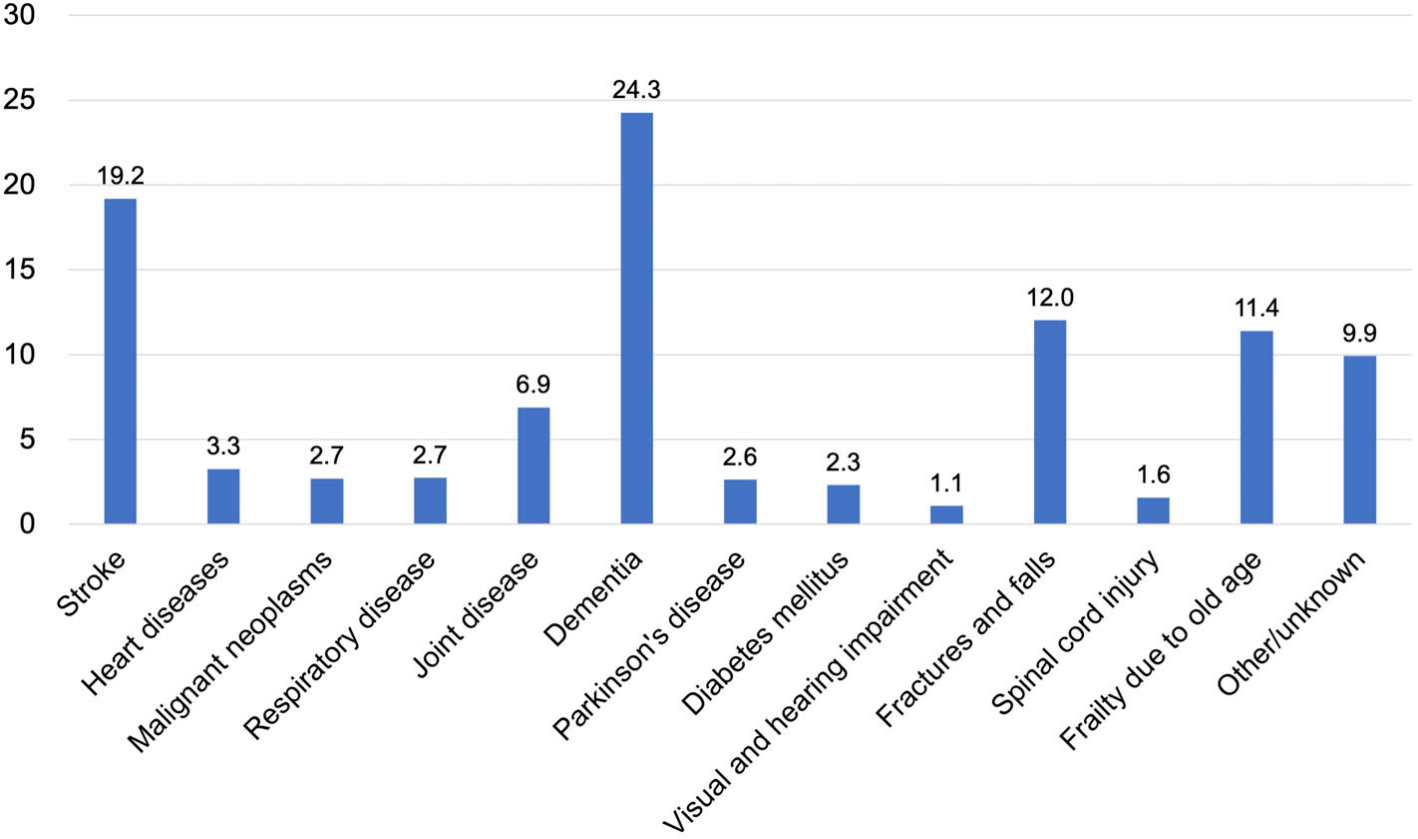
Percentage of major causes of the need for care among those who require care, 2019 National Survey of Family Life. Drawing based on data in [Ministry of Health, Labour and Welfare]^8^ according to the Terms of Use.

Falls can cause fractures or a decline in motor functions to a level that requires nursing care. For those requiring nursing care in Japan, falls and fractures are the third leading cause of requiring nursing care, after dementia and cerebrovascular disease, accounting for 12% of the total (Fig. 3).^8^

**Figure 4:**
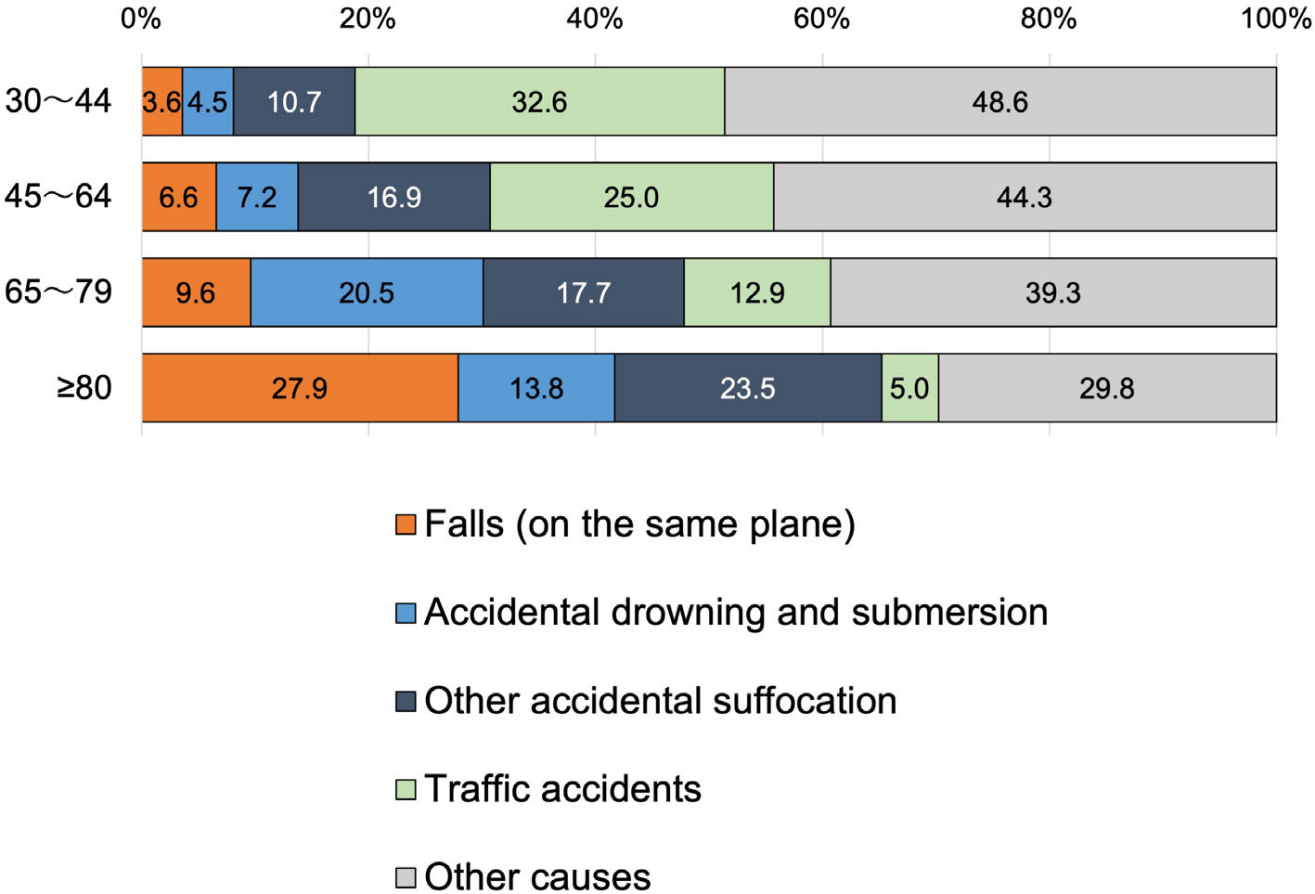
Main causes of death due to unintentional accidents by age group, 2019 Demographics Survey. Among deaths due to unintentional accidents, the proportion of those due to falls increased dramatically among people aged ≥80 years and was much greater than that of deaths due to traffic accidents. The proportion of unintentional deaths due to falls into bathtubs and/or bath drowning at home was also high among older people, exceeding 40% when combined with deaths due to falls among those aged ≥80 years. Drawing based on data in [Ministry of Health, Labour and Welfare]^9^ according to the Terms of Use.

Falls are shown to be a significant cause of death by statistics on causes of death by unintentional accidents (Fig. 4).^9^ Although traffic accidents are the leading cause of death from unintentional accidents among adolescents and younger adults, the proportion declines among older adults, and the incidence of deaths from falls increases rapidly.

**Figure 5:**
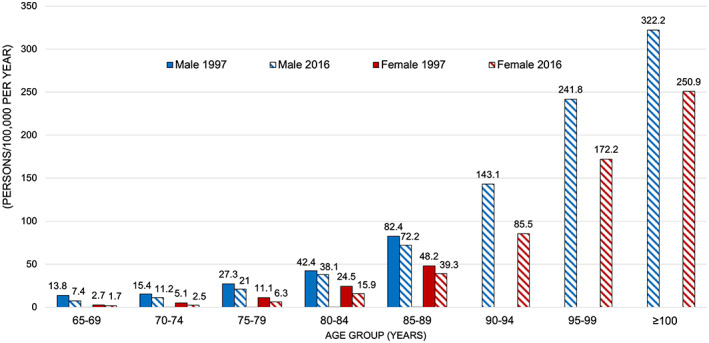
Comparison of fall‐related crude mortality rates by sex and age group, 1997 and 2016. This figure is based on the data in Table 1 of Hagiya *et al*.^10^ Mortality rates in 1997 are not shown in the figure because the data for people aged ≥90 years are grouped, but the total mortality rates in people aged ≥90 years were 159.3 for men and 122.4 for women.

Overall, fall‐related deaths in Japan have continued to decline over time. According to a study that analyzed fall‐related mortality rates by age group for >20 years from 1997 to 2016 in the Vital Statistics Survey of the Ministry of Health, Labor and Welfare of Japan, the mortality rate decreased by 2.5% for men and 2.8% for women in the 65–74 years age group.^10^ In the 75–84 years age group, a decreasing trend was observed, but at a smaller rate, and in the ≥85 years age group, no significant decreasing trend was observed for 20 years for men, and no significant decreasing trend was observed for women after 2006. Figure 5 shows the fall‐related crude mortality rates by age group reported in the study, with values extracted for 1997 and 2016. The exponential increase in fall‐related deaths with age for both men and women is the same for both years. Although fall‐related deaths have decreased in all comparable age groups over the past 20 years, the rate of fall‐related deaths has decreased as age increases. These findings suggest that, although advances in medical and nursing care have reduced the number of fall‐related deaths, such a reduction was inversely related to age. Among men aged ≥80 years and women aged ≥85 years, the percentage reduction in crude mortality was only in the range of 10–20%, even for this 20 years, suggesting that preventable fall‐related deaths are limited in populations with high rates of fall‐related deaths.

These data on the current situation in Japan include residents of facilities, but most of the data refer to community‐dwelling residents. The conditions pertaining to an even higher age group than the equivalent age group in Japan should apply to facility residents requiring a higher level of care and rehabilitation.

## The reality of falls and fall‐related injuries in facilities

Scientific evidence is required to determine the effectiveness of the fall prevention measures recommended for the general older population, because many residents of facilities are not only older in age, but also have numerous and complex factors putting them at risk for falls.

First, we decided to collect studies that prospectively assessed the incidence of falls in residents of facilities to understand the actual situation of fall risk among this population. We used PubMed to search for English‐language articles on falls in those aged ≥65 years, including systematic reviews (SR), meta‐analyses (MA), clinical studies such as randomized controlled trials (RCT), and group comparison studies. Using the following search procedure, 172 articles were extracted (carried out on 28 September 2020).

#1 Set “Search Filters” to “Aged: 65+ years,” “Language: English.”
#2 For facilities, select “long‐term care” [MeSH Terms] OR “nursing homes” [MeSH Terms] OR “intermediate care facilities” [MeSH Terms] OR “skilled nursing facilities” [MeSH Terms]: 28492 publications.
#3 For falls, select “accidental falls” [MeSH Terms]: 13 321 publications.
#4 #2 AND #3: 753 publications
#5 From #4, under “Publication Type Filters,” select “Systematic review/Meta‐analysis/Guideline OR Randomized controlled trial OR Clinical trials OR Comparative studies:” 172 publications.
#6 Two members of the committee independently extracted papers from #5 that show the actual situation regarding the risk of falling for residents of facilities, and the results obtained by the two members were combined: 15 publications.
#7 No additional publications were retrieved while writing the statement or during the peer review process by external committee members.

A summary of the 15 extracted papers is shown in Table 3.^11^, ^12^, ^13^, ^14^, ^15^, ^16^, ^17^, ^18^, ^19^, ^20^, ^21^, ^22^, ^23^, ^24^, ^25^ The following data should be recognized as indicative, because the type of LTCF and the health status of the residents are not necessarily the same in Japan and overseas. Additionally, the health status of the residents and the evaluation method for falls vary among the studies. The residents studied were mainly in the 80–85 years age group, with approximately 70% being women, and the incidence of falls was approximately two times per person per year, with approximately 40% of the residents experiencing at least one fall event. Approximately 10% of the residents experienced a serious fall event in a year, causing a fracture or requiring a transfer to the hospital. In an LTCF with 100 residents, approximately 40 residents fell an average of five times in a year, and approximately 10 of them suffered bone fractures.

**Table 3:** Risk factors for falls in nursing homes

Author, year of publication	Setting, study duration	Residents	Analysis factor	Incidence of falls	Rate of falling	Rate of injurious fall	Risk factors
Damián J, 2013^11^	Observational study	*n* = 733 Age: 83.4 years Women: 76%	General risk factors	2.4/person/year			Multimorbidity, incontinence, antidepressants, arrhythmia, polypharmacy
Becker C, 2006^12^	Observational study, 1 year	*n* = 881 Age: 85.0 years Women: 79.1%	Weekdays and holidays	1.447 on weekdays, 1.193 on holidays/person/year		Femur neck fractures 0.025 on weekdays, 0.030 on holidays per person/year Other fractures 0.016 on weekdays, 0.034 on holidays per person/year	Weekdays/holidays do not affect
Thapa PB, 1998^13^	Observational study	Non‐users of antidepressants, *n* = 847 Users of antidepressants, *n* = 1571 Age: 82 years Women: 75%	Three types of antidepressants	1.31/person/year (non‐users of antidepressants) 2.98/person/year (users of antidepressants)			Antidepressants (no difference between drugs)
van Doorn C, 2003^14^	Observational study, 2 years	Non‐dementia, *n* = 1044 Dementia *n* = 971 Age: 81.4 years Women: 70.4%	General risk factors	2.33/person‐year (non‐dementia) 4.05/person‐year (dementia)			Dementia: RR 1.74 (adjusted RR 1.93) Unsteady gait: RR 1.44 Recent fall history: RR 1.84 Parkinson's disease: RR 2.16 Antipsychotic drugs: RR 1.83 Antidepressants drugs: RR 1.44
Sterke CS,2012^15^	Observational study, average 350 days	*n* = 248 (ambulatory dementia patients) Age: 82 years Women: 59.7%	Psychotropic drugs (including dose–response)	2.9/person/year			Psychotropics, antidepressants, dose–response relationship for these drugs
Buckinx F, 2018^16^	Observational study, 1 year	*n* = 662 Age: 83.2 years Women: 72.5%	Overall risk factors and various fall‐related factors		37.3%/year		Tinetti test, grip strength, isometric elbow extensor strength
Hasegawa J, 2010^17^	Observational study, 6 months	*n* = 1082 Age: 82.5 years Women: 69.8%	Incontinence, behavioral symptoms		24.4%/6 months (48.8%/year)	Non‐traumatic all fracture, injurious falls, hospitalization, etc.: 6.4%/6 months (12.8%/year)	Incontinence: HR 2.14, Behavioral symptoms: HR 1.45
Büchele G, 2014^18^	Observational study, 1 year	70 196 Falls more than 50% of ≥80 years of age Women: 71.0%	General risk factors for falls requiring transfer to hospital			Transfer to hospital: 6.0–9.1%/year	Women: <70 years‐of‐age 6.6, 70s 7.4, 80s 8.6, ≥90 years 9.1%/year Male: <70 years‐of‐age6.7, 70s 6.0, 80s 6.6, ≥90 years 7.7%/year No need for care, ambulatory, slippers use
Thapa PB, 1996^19^	Observational study, 1 year	Ambulatory, *n* = 725 Non‐ambulatory, *n* = 503 40.6% of ≥85 years‐of‐age Women: 76.8%	Ambulatory and non‐ambulatory			Fractures: non‐ambulatory 2.62%/year, ambulatory 11.14%/yearHospitalization: non‐ambulatory 1.66%/year, ambulatory 6.36%/year	
Bronskill SE, 2018^20^	Observational study retrospective	*n* = 7791 (dementia, *n* = 5066) Age: 83.9 years Women: 63.9%	Trazodone and benzodiazepines (90 days after initiation)			Transfer to hospital: 5.74–6.03%/90 days (~24%/year), Transfer to hospital (dementia) 6.36–6.38%/90 days (~26%/year)	
Cox CA, 2016^21^	Observational study, 2 years retrospective	*n* = 2368 Age: 83.9 years Women: 68.9%	Psychotropic drugs, antidepressants, benzodiazepines	1.7 persons/year			Psychotropics, antidepressants, benzodiazepines: OR 2.88 (95% CI 1.52–5.44)
Landi F, 2014^22^	Observational study, 1 year	*n* = 1490 Age: 83.6 years Women: 71.5%	Anticholinergic risk scale				≥80 years‐of‐age 1.69‐fold, ADL decline 1.04‐fold, anticholinergic risk scale 1.26‐fold
Deandrea S, 2013^23^	SR/MA	18 studies Age 80–85 years‐of‐age >75% women	General risk factors				OR ≥2: history of falls 3.06, use of walking aids 2.08, moderate or severe disability 2.08 OR 1–1.99: cognitive impairment 1.73, Parkinson's disease 1.65, dizziness 1.52, no. medications (for 1 drug increase) 1.05, sedatives 1.41, psychotropic drugs 1.61, antidepressants 1.35
Kröpelin TF, 2013^24^	SR	8 studies (dementia)	General risk factors				Psychotropic drugs, physical restraints, health conditions
Sterke CS, 2008^25^	SR	17 studies (dementia)	Psychoactive drugs				Multiple drugs, antidepressants, anti‐anxiety drugs (MA were not conducted due to high heterogeneity of the studies)

CI, confidence interval; HR, hazard ratio; MA, a meta‐analysis; OR, odds ratio; RR, relative risk; SR, systematic review.

The characteristics of individuals prone to falling can also be found in Table 3. A history of falls, use of walking aids and moderate or greater physical disability are of particular importance, followed by the presence of cognitive impairment, Parkinson's syndrome and use of various psychiatric medications. Among factors that can be ameliorated through intervention, it is also important to note that scientific evidence shows that wearing footwear, such as slippers without heel fixation, is dangerous. Falls occur more commonly among people requiring a level of care that allows them to move independently than among those requiring a higher level of care. This is consistent with the fact that falls are more likely to occur when physical functions tend to improve during rehabilitation, and that they can occur in unexpected places within the facilities.

## Scientific evidence on the prevention of falls and fall‐related injuries in facilities

RCTs on fall prevention measures constitute the best scientific evidence on their effectiveness. The Cochrane Database of Systematic Reviews regularly revises its SR titled “Interventions for preventing falls in older people in care facilities and hospitals”.^2^ Table 4 shows a summary of the results.

**Table 4:** Summary of systematic reviews on the effectiveness of fall prevention measures in nursing homes

Intervention	Rate of falls	Risk of falling
Exercise	Unreliable (very low‐quality evidence)	No difference (low‐quality evidence)
General medication review	No difference (low‐quality evidence)	No difference (low‐quality evidence)
Vitamin D supplementation^†^	Probably decrease (moderate‐quality evidence)	No difference (moderate‐quality evidence)
Multifactorial interventions	Unreliable (very low‐quality evidence)	No difference (low‐quality evidence)

A total of 71 randomized controlled trials were included in the analysis, with a total of 40 373 participants, mean age of 83.5 years and 75.3% women. A total of 10 randomized controlled trials included patients with dementia.

^†^ It is estimated that the target population of the included studies had low vitamin D levels. Newly created by the authors based on the description in Cameron *et al*.^2^.

To check whether any changes should be made to the conclusions of this SR, we searched the Cochrane Library and MEDLINE for articles published after September 2017, while excluding hospital‐related search terms by using the same search formula used by Cameron *et al*.^2^ In total, 334 articles were extracted after eliminating duplicates and articles with old publication dates. From these, we selected RCTs that prospectively compared the incidence of falls among older residents, the percentage of those who fell and the effects of injuries (fractures, death etc.) caused by falls (fall prevention and fall injury reduction effects) according to the fall prevention measures used in facilities. We excluded articles on the incidence of falls as an adverse event, because falls were not always defined in advance, and their occurrence as an event was not reliably ascertained, which could lead to inaccurate assessment of falls. The 334 articles were independently selected by two members of the committee, and the results were integrated and finalized by consultation, leading to the final selection of six RCTs (Table 5).^26^, ^27^, ^28^, ^29^, ^30^, ^31^ In addition, five SRs were published after the one by Cameron *et al*.^2^ We examine the differences in the setting of the target population, the year of publication of the selected papers, and the conclusions of Cameron *et al*. and the cited SRs in the following discussion.^32^ No additional papers were identified during the statement preparation process or during the peer review process by external committee members.

**Table 5:** Randomized controlled trials on fall prevention measures in nursing homes (articles published after September 2017)

Author, year of publication	Residents	Intervention	Study design, control	Measurements; time of assessment	Main results
Arrieta, 2019^26^	*n* = 112 Age: 84.9 years Women: 70.5%	Progressive multicomponent exercise at moderate intensity 6 months	RCT Control group: routine low‐intensity activity	Frailty Falls Mortality; 6 and 12 months	After 6 months: The prevalence of frailty was lower in the intervention group (*P* < 0.05). Fewer falls were observed in the intervention group (*P* < 0.05). After 12 months: The decline in ADLs was significant in the control group (*P* < 0.05). There was no difference in the incidence of falls between both groups at 6–12 months. Lower overall mortality was observed in the intervention group (*P* = 0.05).
Hewitt, 2018^27^	No. facilities: 16 *n* = 221 Age: 86 years Women: 65.2%	High level balance and moderate intensity progressive resistance training 6 months	Cluster RCT Control group: usual care	Rate of falls; 12 months	Intervention Usual Care GroupGroup No. residents 113 108 Rate of falling (/person‐year)1.312.91 Total no. falls 142 277 No. falls, ≥5 times920 No. injurious falls72 157 No. emergencies1741 IRR of falls in the intervention group: 0.45 (95% CI 0.17–0.74) No increase in serious adverse events by intervention
Stanmore, 2019^28^	No. facilities: 18 (residential care) *n* = 106 Age: 78 years Women: 78%	Virtual reality (VR) game program for strength and balance training 12 weeks	Cluster RCT Control group: standard community fall prevention advice	Balance ability Rate of falls Fear of falling Pain	Significantly improved balancing ability in the intervention group The fear of falls and pain significantly reduced, and the rate of falls was significantly lower in the intervention group. No. falls: intervention group 20%, control group 24%. Rate of falls: 1.26/person/year in the intervention group, 3.11/person/year in the control group (IRR: 0.31, 95% CI 0.16–0.62, *P* = 0.001) Cost‐effectiveness in fall prevention was significantly better in the intervention group.
Toots, 2019^29^	No. facilities 16 *n* = 186 Age: 85.1 years Women: 75.8% Dementia: a mean Mini‐Mental State Examination score of 15	High‐intensity functional exercise 4 months	Cluster RCT Control group: normal activity	Falls Fall‐related injuries; 12 months after intervention completion	No difference in the rate of falls between both groups There were fewer moderate/serious fall‐related injuries in the intervention group at 12‐months follow‐up (OR 0.31 95% CI 0.10–0.94, *P* = 0.039)
Haines, 2020^30^	No. facilities 15 *n* = 12 548 beds/day Age and women ratio unspecified	Multidisciplinary care plans by GPs, nurses, and care staffs 90 weeks	Cluster RCT Control group: standard model of care	Falls Unplanned Hospital Transfer Polypharmacy	The number of falls increased in the intervention group (IRR 1.37, 95% CI 1.20–1.58). The number of unplanned hospital transfers (IRR 0.53, 95% CI 0.43–0.66) and hospitalizations (IRR 0.52, 95% CI 0.41–0.64), and of out‐of‐hours GP callouts (IRR 0.54, 95% CI 0.36–0.80) decreased in the intervention group.
Mackey, 2019^31^	150 rooms *n* = 357 Age: 81.7 years Women: 64.3%	Resident rooms with low stiffness “compliant” flooring 4 years	RCT Control group: resident rooms with rigid control flooring	Serious fall‐related injuries	There was no difference in the incidence of serious fall‐related injuries between the intervention group and the control group.

CI, confidence interval; IRR, incidence rate ratio; OR, odds ratio; RCT, randomized controlled trial.

Regarding exercise, three studies showed efficacy, whereas one carried out among people with dementia showed no efficacy. Although we did not carry out a meta‐analysis that included previous studies because of the different conditions under which the research papers were extracted, the results of the study by Arietta *et al*., which showed an effect on the onset of frailty and death at 1 year, are noteworthy.^26^ In contrast, a study by Hewitt *et al*. found a reduction in the rate of falls of >50%.^27^ Thus, it is conceivable that the effect of exercise can be changed from “uncertain” to “expected,” supporting the usefulness of rehabilitation in facilities. However, even in such conditions, it should be considered that 1.31 falls per year would still occur.

An SR on the impact of prescription drug review on falls has also been reported.^32^ A meta‐analysis of RCTs comparing a prescription review intervention group with a control group in 18 408 cases, 83% of whom were aged in their 80s, showed a 24% (95% CI 0.62–0.93) reduction in falls and a 26% (95% CI 0.65–0.84) reduction in the total mortality rate.

By summarizing the previously reported SRs and the RCTs published since then, the measures that should be actively implemented to prevent falls are gradually becoming clearer, although the scientific evidence is not sufficient for the older adults in facilities who are the subject of this statement. However, even studies on the efficacy of interventions report the occurrence of many falls that cannot be avoided by preventive measures, simply because the population has a high incidence of falls to begin with. Although it is important to continue to improve fall prevention measures, the current scientific conclusion is that it is reasonable to consider the occurrence of fall‐related fractures and fall‐related deaths as one of the outcomes of geriatric syndromes, even when standard measures, such as environmental maintenance in facilities, are implemented.

## The importance of maintaining and improving the daily living functions of residents in long‐term care facilities while considering the risk of falls: Risk of falls and fall prevention measures for residents of facilities

Many academic organizations, including the World Health Organization, have proposed measures to prevent falls, and there is scientific evidence to support the increasing effectiveness of such measures.^3^, ^4^, ^6^ In fact, the number of fall‐related deaths in Japan has steadily decreased over the past 20 years since 1997, although fall‐related deaths still increase exponentially with age, and the rate of decline in fall‐related deaths for >20 years was smaller in the older age groups.^10^ In other words, it is necessary to consider that older people at extremely high risk of falling have a certain probability of falling and a certain probability of suffering a fracture or dying if they are active, whether in facilities or at home.

Scientific evidence based on RCTs has not yet identified useful interventions that can be recommended as standard measures for fall prevention in facilities. This implies that the effectiveness of fall prevention measures recommended for the general older population is extremely limited in facilities, because many residents are in the age group in which fall‐related deaths increase exponentially and their health status is inferior to that of the general population; therefore, they are subject to many complex and unadjustable individual factors causing falls. Similar considerations might apply to the effectiveness of environmental improvements for fall prevention in the facilities themselves.

## Reducing injury in the event of a fall

The prevention of fall‐related injuries and the maintenance of daily living functions are the fundamental objectives of fall prevention. Fractures and intracranial hemorrhage associated with head trauma are the most common fall‐related injuries that have a significant impact on daily living function and prognosis. Regarding fractures, there is evidence that pharmacotherapy suppresses proximal femur fractures in community‐dwelling and outpatient older people aged ≥80 years.^33^, ^34^, ^35^ Diagnosis and treatment of osteoporosis are desirable.^36^ In particular, for secondary prevention of fragility fractures of the vertebral body or proximal femur, the necessity of medical intervention should be considered even without bone density testing.^37^ Recommendations were made to mitigate the impact of falling from bed when the patient can climb over the bed fence: use of hip protectors, use of shock‐absorbing mats, adjustment of bed height and considering the use of protective caps.^4^ Although the evidence in nursing homes is limited and the usefulness of such interventions is unclear, they should be considered, depending on the situation of the residents and the LTCF.^38^

## Initiatives to maintain and improve daily living functions

Although exercise is an important intervention for older adults, including for rehabilitation purposes, the corresponding increase in activity levels also leads to an increased risk of falls. As a summary of the SR carried out for this statement, it is important to note as scientific evidence that the occurrence of falls did not necessarily increase with exercise interventions. This suggests that exercise interventions that are necessary to maintain individual daily living function and physical activities should be aggressively practiced.

What we would like to emphasize in this statement is that the focus should not only be on falls and fall‐related injuries and deaths, but we should instead continue our efforts to maintain and improve the daily living functions of residents, accepting falls as one of the predicted outcomes. Although it is expected that rehabilitation to improve motor function will result in increased activity and increased chances of falling, we should not forget that the advantages of increased activity outweigh its disadvantages. In addition to fall prevention, some published studies have shown the benefits of exercise interventions on life expectancy, quality of life and prevention of frailty. Proactive interventions should be implemented and continued, to improve as much as possible daily living functions, quality of life and life prognosis. The interventions should not be limited by the risk of falls, which should be controlled by reviewing medications that might increase the risk of polypharmacy and falls, while avoiding behavioral restrictions, including physical restraints, as much as possible, and carrying out interventions, including fall prevention, in a multidisciplinary manner.

## Conclusion

In facilities, as the age of residents and the percentage of people with dementia continue to increase, there is a need to raise the level of care. In addition to the efforts of individual facilities, there are ongoing efforts to promote the development of systems under the long‐term care insurance system and related organizations. As abolishing physical restraints in facilities is a fundamental principle set against the backdrop of the basic philosophy of the Long‐Term Care Insurance Law, which is to maintain dignity and support independence, part of the safety measures to reduce the increased risk of accidents, such as falls in such situations, is described below.

Under the long‐term care insurance system, in addition to mandatory training in various areas, such as dementia, from 2021 it will be mandatory for facilities to appoint a person in charge of safety measures, and it is expected that organizational safety measures will be further developed. In contrast, even in situations where such efforts are being made, it has already been noted in this statement that inevitable falls and related injuries might still occur.

This statement scientifically examines, to the extent that it is possible to grasp, the balance between the attitude and efforts of facilities to prevent falls and associated injuries, and the mental representation of residents, their families and the public, who accept that accidents will occur under the circumstances. To maintain this balance, it is important to reiterate the importance of information sharing and mutual understanding regarding the occurrence and prevention of falls between facilities and the residents themselves and their families. In addition, although this statement focuses on residents of facilities, the same concept applies to older people using daycare facilities.

With the advancement of nursing care technology, scientific analysis based on databases on nursing care safety has been initiated. It is becoming possible to learn about fall prevention not only from cases of falls, but also from cases of prevention in residents at high risk of falling. Furthermore, the development of science and technology, including artificial intelligence, is expected to lead to the evolution of fall prevention behaviors based on prediction and new safety equipment installed in facilities. Although not included in this statement, academic organizations and organizations involved in nursing care, as well as industry and government, must continue to work to advance the science of nursing care, including fall prevention, and establish systems that are able to incorporate such science.

## Disclosure statement

The authors declare no conflict of interest.

## Data Availability

Data sharing is not applicable to this article as no new data were created or analyzed in this study.
